# Clinical implications of AGBL2 expression and its inhibitor latexin in breast cancer

**DOI:** 10.1186/1477-7819-12-142

**Published:** 2014-05-07

**Authors:** Hao Zhang, Yuan Ren, Deyan Pang, Caigang Liu

**Affiliations:** 1 Department of Breast Surgery, Second hospital of Dalian Medical University, Zhongshan Road, Dalian 116023, People’s Republic of China; 2 Department of Hematology, First hospital of China Medical University, Nanjing Street, Shenyang 110001, People’s Republic of China; 3 Department of Mathematics, Northeast Yucai School, Shiji Road, Shenyang 110179, People’s Republic of China

**Keywords:** Breast cancer, Cancer stem cell, AGBL2, latexin, Survival

## Abstract

**Background:**

We investigated the expression status of AGBL2 and its inhibitor *latexin* in breast cancer stem cells and its clinical implications in order to lay a foundation for managing breast cancer.

**Methods:**

CD44+/CD24- tumor cells (CSC) from clinical specimens were sorted using flow cytometry. AGBL2 expression status was detected in CSC and 126 breast cancer specimens by western blot and immunohistochemistry staining. The relationship between the AGBL2 protein and clinicopathological parameters and prognosis was subsequently determined.

**Result:**

As a result, CSC are more likely to generate new tumors in mice and cell microspheres that are deficient in non-obese diabetic/severe combined immunodeficiency mice (NOD/SCID) compared to the control group. The AGBL2 protein was expressed higher in CSC induced to epithelial to mesenchymal transition (EMT) when compared to the control cells, and was found to be related to CSC chemotherapy resistance. After Spearman regression correlation analysis, AGBL2 was observed to be related to clinical stage, histological stage, and lymph node metastasis. In the Cox regression test, the AGBL2 protein was detected as an independent prognostic factor. Through immunoprecipitation, AGBL2 and *latexin* could form immune complexes.

**Conclusions:**

These results demonstrate that AGBL2 is a *latexin-*interacting protein that regulates the tubulin tyrosination cycle and is a potential target for intervention.

## Background

Breast cancer is the most common cause of death of all female malignant tumor diseases [1]. In 2008, 1,380,000 new occurrences of breast cancer were diagnosed worldwide, with 458,400 persons dying from breast cancer that same year [2,3].

Stem cells, which represent only a very small percentage of the total tumor mass, have been found to be the source of some, and possibly most, cancers [4]. Breast cancer stem cells are a small group of tumor cells with the capacity to self-renew, a strong ability to form solid breast tumors, and the ability to differentiate into a relatively quiescent primitive group of cancer cells that are considered the underlying factor of tumor recurrence and the main reason that breast cancers resist therapies [5].

AGBL2 is a cytoplasmic carboxypeptidase. Knockdown of AGBL2 results in a failure of the cell to detyrosinate the C-terminal EEY region of α-tubulin, and indicates that it is a candidate for the long sought after tubulin tyrosine carboxypeptidase important in regulation of microtubule dynamics [6]. Retinoic acid receptor responder 1 (*RARRES1*), also known as tazarotene-induced gene 1 (*TIG1*), was first identified as a novel retinoid-responsive gene in skin. Notably, a *RARRES1* family member, *latexin*, was initially described as the only known mammalian carboxypeptidase inhibitor and is involved in the regional specification of neurons [7-10]. Despite extensive evidence for the tumor suppressor role of *RARRES1*, no mechanism for its biological function has been determined.

AGBL2 is reported to be involved in tumor epithelial to mesenchymal transition (EMT), but the mechanism by which it operates is still unclear [6]. Currently, the AGBL2 expression status in breast cancer stem cells (CSC) and the clinical implications for breast cancer are also unclear. In the present study, we try to sort and identify breast cancer stem cells investigate the expression status of AGBL2 in those cells, and evaluate the clinical implications of AGBL2 in breast cancer. Gaining this knowledge will lay a foundation for managing breast cancer.

## Methods

### Patients and tissue specimens

A total of 126 patients who had histologically confirmed breast cancer and who underwent radical operations in China Medical University between January 2001 and January 2006 were enrolled for immunohistochemical and immunofluorescence double staining and prognostic analysis. The mean age was 50.73 ± 10.28 years (range from 27 to 80 years). The criteria to include a patient in this study were as follows: (1) curative operations (modified radical mastectomy plus axillary lymph node dissection) were performed; (2) resected specimens were pathologically examined; (3) more than 10 lymph nodes were pathologically examined after the operation; and, (4) a complete medical record including the estrogen receptor (ER), progesterone receptor (PR), human epidermal growth factor receptor 2 (Her2), tumor protein P53 (p53), and antigen Ki67 (Ki67) status was available. The study protocol was approved by the Ethics Committee of China Medical University and written informed consent was obtained from all participants involved in the study.

### Identifying the ability of cancer stem cells to form tumors

The clinical specimens were digested into single tumor cells using collagenase III (Boster, Wuhan, China). The tumor cells were suspended in 100 μl/10^6^ cells of Hank’s balanced salt solution (HBSS) with 2% heat-inactivated calf serum (HICS). The samples were then washed twice with HBSS/2% HICS and suspended. Antibodies, including anti-CD2, -CD3 -CD10, -CD16, -CD18, -CD31, and anti-CD326 were added and incubated on ice for 20 minutes and then washed twice with HBSS/2% HICS. Lineage + cells were first eliminated using anti-CD2, -CD3 -CD10, -CD16, -CD18, -CD31, and anti-CD326 during flow cytometry. Dead cells were eliminated using the viability dye 7AAD (Boster, Wuhan, China). Next, CD44+/CD24- tumor cells were sorted by CD44 and CD24 in flow cytometry. The selected cells to be injected were then suspended in a 1640/Matrigel mix (1:1 volume) (Boster, Wuhan, China) and injected into the appropriate area of the mammary fat pad.

### Mammosphere generation test

For this step, complete MammoCult™ medium (human) (Boster, Wuhan, China) was prepared by adding 50 mL of thawed MammoCult™ proliferation supplements (human) (Boster, Wuhan, China) to 450 mL of MammoCult™ basal medium (human). Single cells were plated on ultralow attachment plates (Corning, Acton, Massachusetts, United States) in the complete MammoCult™ medium at a density of 20,000 viable cells/mL. The number of spheres for each well was evaluated after seven days of culture.

### siRNA transfection and the effect confirmation

AGBL2 siRNA and control siRNA were designed and synthesized by Wanlei Biotechnology, Inc (Shenyang, China). The downregulation of AGBL2 by siRNA was confirmed by RT-PCR and western blot at the indicated time points.

### Transfection and stable colony selection

Transfection was performed using the transfection reagent Lipofectamine™ 2000 (Invitrogen) (Boster, Wuhan, China) based on the manufacturer’s instructions. The clones derived from breast cancer cells stably expressing *latexin* were selected for further experiments.

### Treatments with chemotherapeutic agents and measuring cell viability

When the above cells, cultured as monolayers, were healthy and were 80 to 90% confluent, they were washed with warm HBSS. The cells were scraped gently from the dish using a sterile cell scrape. The scraped cells were then resuspended in complete MammoCult medium and counted. The sensitivity of the cells to three chemotherapeutic drugs were examined using the cell counting kit-8 (CCK-8) (Boster, Wuhan, China) technique. Cells were plated at a density of 5 × 10^4^/mL cells per well into ultralow attachment 96-well plates containing 100 μl complete MammoCult medium and treated with a concentration of cisplatin (DDP) (2.5 μg/ml/PPC (plasma peak concentration)), epirubicin (EPI) (0.78 μg/ml/PPC), and docetaxel (DTX) (3.7 μg/ml/PPC) as follows: 0.2, 1.0, 5.0, 10.0 PPC. CCK-8 reagent was added to each well and incubated for two hours before reading at wavelength of 450 nm. The cells were counted at 48 and 72 hours with CCK-8.

### Western blot analysis

For western blot analysis, the cells were lysed with the buffer [0.1% sod dodecyl sulfate (SDS), 50 mmol/L Tris Hydrochloride (Tris–HCl) (ph 7.6), 1% NP-40, 150 mmol/L NaCl, 2 mg/ml aprotinin, 2 mg/ml leupeptin and 7 mg/ml phenylmethanesulfonyl fluoride (PMSF)]. The protein concentrations were determined using the bicinchoninic acid (BCA) protein assay kit (Pierce Biotechnology Inc., Rockford, Illinois, United States). Thirty micrograms of protein were separated on 10% SDS-PAGE gels and transferred to a polyvinylidene fluoride (PVDF) membrane. After blocking, the membrane was incubated with anti-AGBL2 antibody (1:500, Biorbyt Ltd., Cambridge, United Kingdom) at 4°C overnight. After washing, the membrane was incubated with a secondary antibody at a dilution 1:2,000 at room temperature for one hour. Proteins were detected with the electrochemiluminescence (ECL) kit (Varsal Instruments, Beijing, China), and anti-β-actin antibody (Sigma-Aldrich, St. Louis, Missouri, United States) was used as a loading control. Densitometry was performed by Gel-pro Analyze software (Media Cybernetics, Silver Spring, Maryland, United States).

### Immunohistochemistry experimental procedures

To score AGBL2 as immuno-positive staining, the positive cells appeared as a yellow to brown color in the nucleus and/or cytoplasm. AGBL2 expression was classified qualitatively according to intensity of staining and semi-quantitatively according to the following criteria: mild/0 if <1% of neoplastic cells discretely expressed AGBL2; intermediate/1+ if ≥1 and <10% of morphologically unequivocal neoplastic cells discretely expressed AGBL2; and strong/2+ if ≥10% of morphologically unequivocal neoplastic cells discretely expressed AGBL2. Samples stained intermediate or strong or scored as 1+ or 2+ were considered positive.

### Tumor growth factor-β1 (TGF-β1) stimulation

Breast cancer stem cells were plated at 4 × 10^4^ cells per 35 mm cell culture dish, and 10 ng/ml recombinant human tumor growth factor-β1 (TGF-β1) (R&D Systems, United States) or vehicle (4 mM HCl and 0.1% albumin from bovine serum (BSA)) was added to the media. Cells were passaged after three days in culture, and TGF-β was replenished. Cells were stimulated for a total of six days before using.

### Immunoprecipitation

Breast cancer cells were lysed using a radio-immunoprecipitation assay (RIPA) buffer and centrifuged at 14,000 × g for 15 minutes. The supernatant was recovered and pre-cleared by adding 1 μg of normal immunoglobulin G (IgG) premixed with 20 μL of A/G protein-bead slurry. The mixture was incubated for 30 minutes at 4°C and then centrifuged at 1,000 × g for 5 minutes. The supernatant was recovered and mixed with 10 μL of primary antibody and incubated for 1 hour at 4°C. A total of 20 μL of A/G protein-bead slurry was then added and incubated at 4°C for 1 hour. Samples were then centrifuged and supernatants were discarded. The precipitate was boiled for 3 minutes after adding 20 μL of SDS-PAGE sample buffer to release the complex from the beads. Western blotting was then performed as described above.

### Statistical analysis

All data were analyzed with SPSS Statistics software (Version 13.0, Chicago, Illinois, United States). Relationships between AGBL2 and other parameters were studied using the chi-square test, Fisher’s extract test, or independent *t* tests. Disease-specific survival was analyzed using the Kaplan-Meier method. The log-rank test was used to analyze survival differences. Multivariate analysis was performed using the Cox proportional hazards model selected in forward stepwise. A *P* value of less than 0.05 was considered statistically significant.

## Results

### AGBL2 expression in breast cancer and its relationship with clinicopathological characteristics

**Figure 1:**
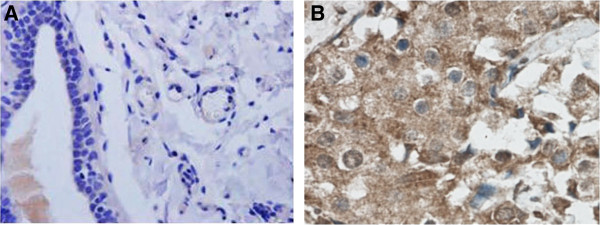
AGBL2 protein was expressed significantly higher in breast cancer tissues (B) compared to paracancerous tissue and atypical hyperplasia tissues (A).

Breast cancer tissues, paracancerous tissues, and atypical hyperplasia tissues were all from the same patients. Tissue samples were stained with hematoxylin and eosin to determine the histological type and tumor grade. Immunohistochemical examination showed that AGBL2 was located in the cytoplasm and membrane of the breast cancers (Figure 1). It was also observed that the AGBL2 protein was expressed significantly higher in breast cancer tissues when compared to paracancerous and atypical hyperplasia tissues (28.63% versus 4.32% versus 2.91%, respectively) (Figure 1A and 1B). The cases with high AGBL2 expression tended to develop into lymph node and postoperative distant metastasis (Table 1).

**Table 1:** Correlations between AGBL2 expression and clinicopathological features (n = 126)

**Variables**	**N**	**AGBL2**^**-**^	**AGBL2**^**+**^	***P *** **value**
**Age**				0.062
<35 Y	18	14	4	
>35 Y	108	60	48	
**Tumor size**				0.562
T1	24	16	8	
T2	83	46	37	
T3	19	12	7	
**Clinical stage**				0.001
I	15	14	1	
II	57	45	12	
III	54	15	39	
**Tumor stage**				0.151
DCIS	16	7	9	
IDC	110	67	43	
**Metastatic nodes**				0.006
Negative	50	38	12	
Positive	76	36	40	
**Her2 status**				0.632
Positive	21	9	12	
Negative	105	47	58	

Spearman correlation regression analysis showed that AGBL2 expression has a linear correlation to histological stage, lymph node metastasis, and clinical stage (*P* = 0.036, 0.001, and 0.007, respectively) (Table 2). After multivariate analysis, the clinical stage, lymph node metastasis, tumor size, and AGBL2 expression were related to postoperative distant metastasis (*P* = 0.001, 0.000, 0.003, and 0.031, respectively) (Table 3).

**Table 2:** Spearman correlation analysis between clinicopathological features and AGBL2

**Clinicopathological features**	**AGBL2 expression (*P *; Spearman correlation)**
Age	0.076 (0.071)
Tumor size	0.053 (0.083)
Clinical stage	0.007 (0.157)
Histological stage	0.036 (0.121)
Lymph node metastasis	0.001 (0.184)
Her2 status	0.086 (0.036)

**Table 3:** Multivariate analysis of the factors related to postoperative distant metastasis

**Characteristic**	**Exp(B)**	**95% CI for Exp(B)**	***P *** **value**
Age	0.553	0.267-2.292	0.371
Tumor size	1.978	1.176-4.126	0.003
Clinical stage	3.936	1.774-6.882	0.001
Tumor stage	2.217	0.557-7.826	0.072
Lymph node metastasis	4.376	2.287-8.762	0.000
Her-2 status	1.549	0.736-3.472	0.059
AGBL2	1.517	1.073-3.432	0.031

### Prognostic analysis

**Figure 2:**
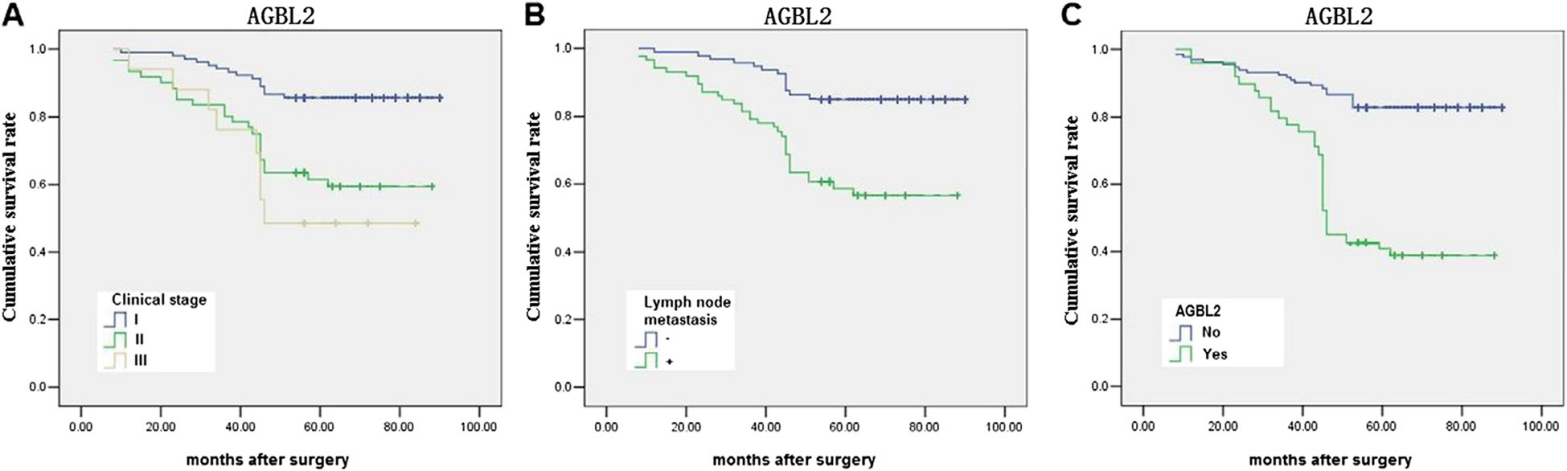
Survival curves about clinical stage (A), lymph node metastasis (B), and AGBL2 expression (C).

The prognostic analysis showed that AGBL2, along with clinical stage, and lymph node metastasis, were significantly associated with a poorer disease-specific survival (*P* = 0.007, 0.002, and 0.004, respectively) (Figure 2).

### Effects of AGBL2-siRNA or latexin on proliferation and invasiveness of breast cancer cells

The methylthiazolyldiphenyl-tetrazolium bromide (MTT) analysis result showed that the proliferation of breast cancer cells was significantly inhibited by AGBL2-siRNA.

We next examined the effect of AGBL2-siRNA and *latexin* on invasion in breast cancer cells using the transwell chamber assay. Our results showed that the invasiveness of breast cancer cells was decreased.

### AGBL2 downregulation or latexin upregulation sensitizes to chemotherapy drugs in cancer stem cells

**Figure 3:**
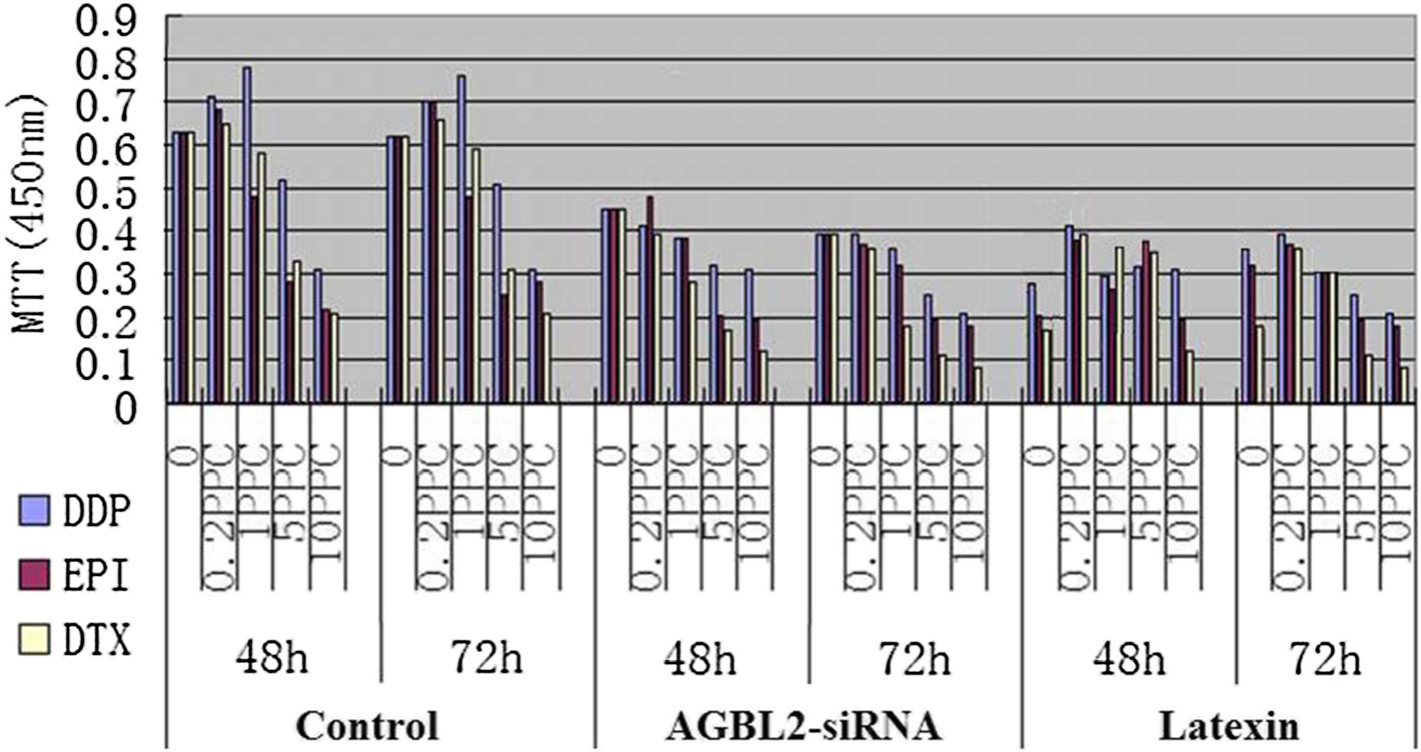
Cells exposed to AGBL2-siRNA or Latexin decreased among the three drugs when compared with the control (DDP: cisplatin, EPI: epirubicin, DTX: docetaxel, MTT: methylthiazolyldiphenyl-tetrazolium bromide, h: hours).

To investigate whether downregulation of AGBL2 expression or upregulation of *latexin* expression has the potential to sensitize CSC to chemotherapy, a combination treatment of AGBL2-specific siRNA or upregulation of *latexin* with chemotherapy drugs was performed. Twenty-four hours after transfection with siRNA, cells were treated with DDP, EPI, and DTX at 0, 0.2 PPC, 1 PPC, 5 PPC and 10 PPC scaled concentrations for 48 and 72 hours. The half maximal inhibitory concentration (IC50) was determined by the MTT assay. Figure 3 shows that the cells exposed to AGBL2-siRNA or *latexin* showed a significant decrease in IC50 among the three drugs when compared with the control (*P* <0.01).

### AGBL2 and latexin expression in breast cancer stem cells that have been induced to EMT

By microarray analysis, we analyzed ten cases, including breast cancer stem cells induced to EMT by TGF-β1 and those not induced. We found that the expression level of AGBL2 significantly increased in breast cancer stem cells induced to EMT, as opposed to those that were *latexin* decreased.

### Identification of the interactome between AGBL2 and latexin

**Figure 4:**
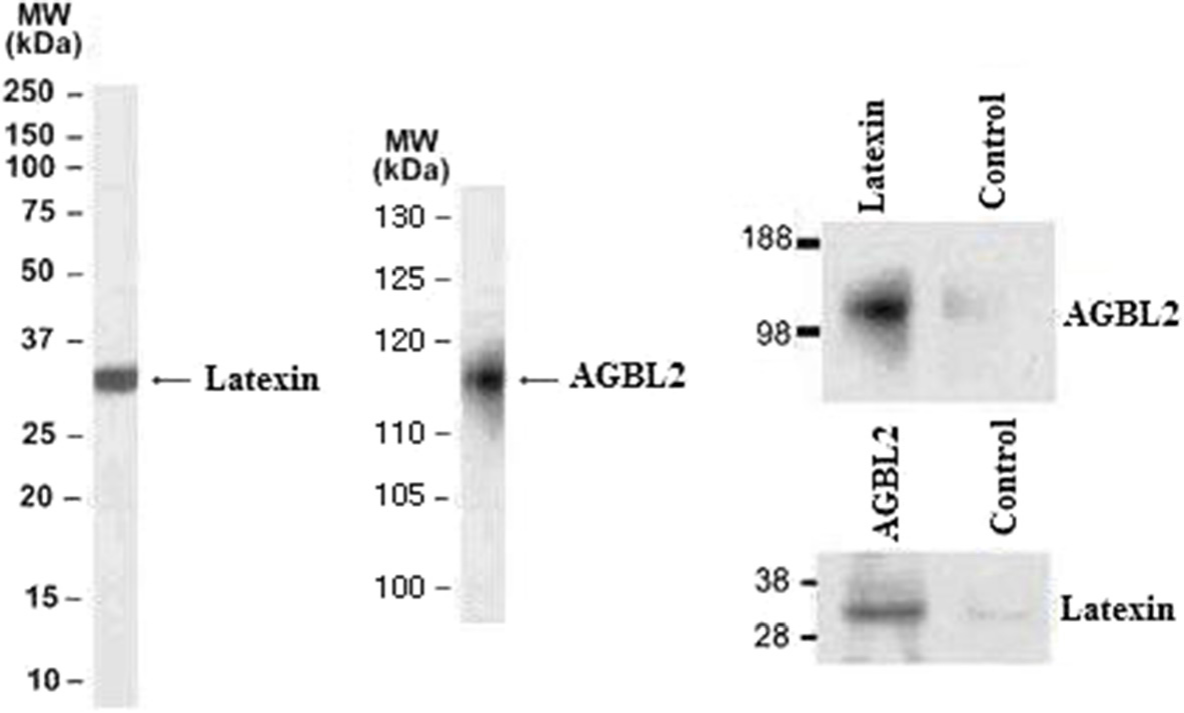
AGBL2 and Latexin could form immune complexes through immunoprecipitation (MW: Molecular Weight).

The presence of AGBL2 in the exogenously- and endogenously-expressed *latexin* complex was confirmed using western blot after tandem affinity purification (TAP), *latexin* immunoprecipitation, and reverse AGBL2 immunoprecipitation. The immunoblot showed the presence of *latexin* and AGBL2 present in the *latexin*-pGlue complex after tandem affinity purification, *latexin* endogenous immunoprecipitation, and AGBL2 endogenous immunoprecipitation (Figure 4). These results demonstrate that AGBL2 is a *latexin-*interacting protein that regulates the tubulin tyrosination cycle and that it is a potential target for intervention.

## Discussion

Tumor stem cells have been found to be the source of most cancers and the culprit of tumor recurrence, metastasis, and drug resistance [11]. In a recent study, AGBL2 was reported as a bridge between cancer stem cell and metastasis [6]. No studies to date, however, have examined the relationship among AGBL2 expression status and breast cancer chemotherapy sensitivity, and the clinical implications of breast cancer. In the current study, we sorted and identified the breast cancer CSC from clinical specimens, observing that AGBL2 was highly expressed in breast cancer CSC induced to EMT when compared to the control group. Moreover, drug sensitivity tests showed that a combination treatment of AGBL2-specific siRNA with chemotherapy drugs could significantly increase the apoptosis of breast cancer CSC. The outcome demonstrated that AGBL2 plays an important role in breast cancer’s resistance to chemotherapy.

We also investigated the relationship between AGBL2 expression and the biological behavior of breast cancer CSC and the clinicopathological characteristics of breast cancer. The AGBL2 protein was observed to be expressed significantly higher in cancerous tissues than tumor-adjacent tissues. Moreover, AGBL2 protein was found to be related to clinical stage, histological stage, and lymph node metastasis.

After survival analysis, AGBL2 was shown to attain a significantly poorer postoperative disease-specific survival. Indeed, the Cox regression test showed that the AGBL2 protein was detected as an independent prognostic factor. These outcomes suggest that AGBL2 is associated with breast cancer CSC.

In a previous study, Ke *et al.*[12] identified high levels of *Latexin* expression in an immortalized human gastric epithelium cell line, GES-1, as compared to expression in the MNNG-transformed GES-1 cells (MC) cell line, which is the malignant derivative of the GES-1 cell line. These findings suggest that downregulation of *latexin* expression is correlated with malignant transformation of immortalized human gastric epithelial cells.

Here, we demonstrated that AGBL2 may also play a role in breast cancer metastasis and may be a potential biomarker for the metastasis and chemotherapy resistance of breast cancer.

## Conclusions

The present study found that AGBL2 was highly expressed in CSC and could be a potential biomarker for the lymph node metastasis and chemotherapy resistance of breast cancer tumors. The underlying genetic mechanism of AGBL2 and its inhibitor latexin in regulating the breast cancer CSC is still unclear and needs further investigation.

## Abbreviations

BCA: bicinchoninic acid; BSA: albumin from bovine serum; DDP: cisplatin; DTX: docetaxel; ECL: electrochemiluminescence; EPI: epirubicin; EMT: epithelial to mesenchymal transition; ER: estrogen receptor; HBSS: Hank’s balanced salt solution; Her2: human epidermal growth factor receptor 2; HICS: heat-inactivated calf serum; IC50: half maximal inhibitory concentration; IgG: immunoglobulin G; Ki67: antigen Ki67; MC: MNNG-transformed GES-1 cells; MTT: methylthiazolyldiphenyl-tetrazolium bromide; NOD/SCID: non-obese diabetic/severe combined immunodeficiency mice; p53: tumor protein P53; PMSF: phenylmethanesulfonyl fluoride; PPC: plasma peak concentration; PR: progesterone receptor; PVDF: polyvinylidene fluoride; RIPA: radio-immunoprecipitation assay; SDS: sod dodecyl sulfate; TAP: tandem affinity purification; TGF-β1: tumor growth factor-β1; Tris–HCl: Tris Hydrochloride.

## Competing interests

The authors declare that they have no competing interests.

## Authors’ contributions

HZ and YR carried out the molecular genetic studies, and drafted the manuscript. DP participated in the design of the study and performed the statistical analysis. CL conceived of the study, and participated in its design and coordination and helped to draft the manuscript. All authors read and approved the final manuscript.
